# The relationship between problematic cell phone use, eating disorders and social anxiety among university students

**DOI:** 10.12669/pjms.37.4.4124

**Published:** 2021

**Authors:** Ayse Gokce, Ali Ozer

**Affiliations:** 1 Dr. Ayse Gokce Bingol Provincial Health Directorate, Bingol, Turkey; 2 Dr. Ali Ozer Department of Public Health, Inonu University, Faculty of Medicine, Malatya Turkey

**Keywords:** Eating disorders, Problematic cell phone use, Students, Social anxiety

## Abstract

**Objective:**

Problematic cell phone use is common among young age groups which include university students, and may be accompanied by social anxiety and eating disorders. We aimed to examine the relationship between problematic cell phone use, social anxiety and eating disorders among university students.

**Methods:**

The universe of this cross-sectional study consists of 28,669 students receiving education at a Inonu University between October 2017 - November 2017. With a confidence interval of 95% and power of 80%, the sample size was calculated to be 308. The survey forms used in the study included students’ sociodemographic characteristics, data regarding cell phone usage, Problematic Mobile Phone Use Scale, Liebowitz Social Anxiety Scale and Eating Attitudes Test. The statistical analyses were conducted using Student t, One Way ANOVA, Spearman Correlation Test and Binomial Logistic Regression Analysis.

**Results:**

The students in the study group demonstrated a 46.1% rate of problematic cell phone use. The students’ Problematic Mobile Phone Use Scale total scores showed a significant correlation with smoking, and daily duration and purpose of cell phone use (p<0.05).

**Conclusion:**

University students demonstrate high rates of problematic cell phone use; in addition, individuals who use cell phones for increased hours or for certain purposes display higher total scores on the Problematic Mobile Phone Use Scale. Students should be educated on limiting problematic cell phone use.

## INTRODUCTION

Due to the rapid advancement in technology throughout the prior years, cell phones have become essential for daily tasks both in our country and around the world. The use of cell phones has become embedded in daily life as they offer numerous benefits for daily efficiency; however, they can be harmful and result in potentially disturbing behaviors, some in which the mechanism of action is not yet clear. According to the 2018 report of the Turkish Statistical Institute (TSI), 97.8% of households contained smartphones in the year 2017, with this rate reaching 98.7% in 2018.^1^ There are very few households that do not contain a smartphone. Besides being appealing and user-friendly, smartphones can be detrimental for the user if used compulsively or problematically. In light of previous studies, it is said that cell phone addiction and problematic cell phone use have a strong correlation and similar concepts are used interchangeably in the literature.^2^,^3^

Problematic cell phone use is defined as the inability of an individual to limit their cell phone use despite the detrimental effects it increases.^4^ Many studies have reported the widespread problematic cell phone use among university students to be an increasingly significant public health concern that causes a decline in the functionality of students due to addiction. In addition, the relationship between problematic cell phone use and social anxiety has been examined in several studies. However, there are limited studies on the correlation between problematic use, social anxiety and eating disorders. The goal of this study was to examine problematic cell phone use and its relationship with social anxiety and eating disorders among university students.

## METHODS

This cross-sectional study was conducted between October 2017 - November 2017 with written permission from the Scientific Research and Publication Ethics Committee (2017\24-11) of Inonu University and the Inonu University Rectorate. The study included students receiving education at Inonu University who were over the age of 18 and agreed to participate. The study universe includes 28669 students. With a confidence interval of 95% and power of 80%, the sample size was calculated to be 308 on the basis of a 28.2% problematic cell phone use rate in the reference study.^5^ The students were first categorized according to their educational faculty and participants from every faculty were then picked using the simple random sampling method. A face-to-face survey was conducted with 104 males and 215 females for a total of 319 people.

The survey form used in the study consisted of four sections. The first section included the students’ sociodemographic characteristics and data regarding cell phone use; the second section consisted of the Problematic Mobile Phone Use Scale (PU); the third section included the Liebowitz Social Anxiety Scale (LSAS); the last section involved the Eating Attitudes Test (EAT).

### Statistical methods

Software programs used for the statistical analysis of data were SPSS 22.0 and “biostatapps”, an artificial intelligence and statistics based interactive web program.^6^ The dependent variables of the study are scores from the PU, LSAS, and EAT, while the independent variables include age, gender, socioeconomic status, place of residence, current residence, and duration of cell phone use. Student t, One Way ANOVA, Spearman Correlation Test and Binomial Logistic Regression Analysis were utilized in the statistical analysis of data and p<0.05 was considered significant in all evaluations.

## RESULTS

The age range of the participating students was 18-33, and their age mean is 21.03±2.05. Table-I displays the sociodemographic distribution of the students participating in the study.

**Table-I:** Sociodemographic Distribution of Students Participating in the Study.

Sociodemographic Characteristics	n	%
***Gender***		
Male	104	32.6
Female	215	67.4
***Faculty***		
Health Sciences	72	22.6
Social Sciences	156	48.9
Science	91	28.5
***Current Residence***		
Government/Private Dormitory	106	33.2
House with Friends/Alone	45	14.1
Family Home	168	52.7
***Region of Longest Residence in Lifetime***		
City	231	72.4
District/Village	88	27.6
Total	319	100

The average daily duration of cell phone use in the study group: 21.6% three hours or less, 31.7% between 4-5 hours, 18.5% between 6-7 hours, and 28.2% longer than eight hours. When asked for their purpose of cell phone use, the students stated that: 55.5% use it to access social media platforms such as Facebook and Twitter, 31.7% use it for WhatsApp, 22.9% for phone calls, 15.7% for SMS messaging, and 11.3% for playing mobile games.

Students who used their cell phones for a longer duration scored significantly higher PU scores (p=0.0001). In the comparison of total PU scores according to purpose of cell phone use; students who use their phone to access social media platforms such as Facebook/Twitter or to play mobile games scored significantly higher compared to those who did not (p<0.05).

The results of the regression test in which the presence of problematic cell phone use in students was assessed with respect to faculty, region of longest residence in lifetime, and presence of an eating disorder or social anxiety is shown in Table-III. Problematic cell phone use was 2.09 times more common in students who spent most of their lifetime in a district or village compared to those who spent it in a city. According to Table-IV, there is a mild, significant, positive correlation between the PU and LSAS scores of the students participating in the study (p=0.001; r=00187).

**Table-II:** Comparison of the Students’ Total PU Scores with Respect to Various Variables

Variables	PU Total Score	p
***Average Duration of Daily Cell Phone Use***		
≤3 hours	29.11±11.64	
4-5 hours	33.93±12.31	0.0001*
6-7 hours	38.42±11.68	
≥8 hours	42.53±13.08	
***Cigarette Use***		
Yes	39.17±14.84	0.024**
No	35.24±12.41	
***Alcohol Use***		
Yes	38.86±14.20	0.098**
No	35.60±12.81	
***Purpose of Cell Phone Use is to Access Social Media Platforms such as Facebook/Twitter***		
Yes	37.60±13.03	0.026**
No	34.32±12.97	
***Purpose of Cell Phone Use is WhatsApp***		
Yes	36.75±12.24	0.374**
No	35.44±14.00	
***Purpose of Cell Phone Use is Phone Calls***		
Yes	34.44±12.12	0.114**
No	36.93±13.46	
***Purpose of Cell Phone Use is Mobile Gaming***		
Yes	40.19±13.45	0.049**
No	39.63±12.97	

* One Way ANOVA

** Student T Test.

**Table-III:** Binomial Logistic Regression Analysis of Independent Variables Associated with the Presence of Problematic Cell Phone Use.

	OR	95% Cl	*p*
***Faculty***			
Health Sciences	1		
Social Sciences/Sciences	1.095	0.607-1.976	0.224
***Region of Longest Residence in Lifetime***			
City	1		
District/Village	2.092	1.245-3.548	0.006
***Eating Behavior Disorder***			
Not present	1		
Present	1.535	0.785-3.04	0.212
***Presence of Social Anxiety***			
Not present	1		
Present	1.427	0.785-3.04	0.212

**Table-IV:** Comparison of PU LSAS and EAT Scores of the Students Participating in the Study.

	LSAS Score	EAT Score
***PU Score***		
r	0.187	0.092
*p*	0.001	0.100
***LSAS Score***		
r	1.00	0.261
*p*	-	0.0001

## DISCUSSION

Twenty-eight-point two percent of students stated that their cell phone usage was eight hours or more. In a 2013 study done with students from a faculty of education, it was found that 34.3% of students used their cell phones for three hours or more daily.^7^ Alongside the fact that the duration of cell phone use is associated with problematic use and addiction, it has been observed that application-oriented use is also significant in leading to problematic use.^8^,^9^ The fact that 28% of the students in our study use their cell phones for eight hours or more may reveal risks associated with problematic use and cell phone addiction.

The rate of problematic cell phone use in the study group students is 46.1%. In the literature, several studies have reported problematic cell phone use rates between 20.1-48.7%.^10^-^13^ A study comparing problematic cell phone use between age groups found that the 16-25 age group had the highest rate of problematic use.^14^ The rate of problematic cell phone use can vary according to the age group, gender ratio and sociocultural differences of the population participating in the study.

In the comparison of PU scores according to cell phone use duration, students who used their phones for a longer duration exhibited significantly higher PU scores. Similar studies have found that the longer the use of mobile phones, the more risk of mobile phone addiction and problematic use are.^15^,^16^ Since the duration of cell phone use induces addiction and problematic use, it is believed that most students in the study group face this risk.

The PU scores of students in the study were significantly higher in smokers compared to non-smokers. While some studies found that smokers exhibited significantly higher rates of cell phone addiction, others did not find a significant relationship between smoking status and cell phone addiction.^15^,^16^

In the comparison of total PU scores according to cell phone use purpose; it was found that students using their phones to access social media platforms such as Facebook/Twitter or playing mobile games had significantly higher scores compared to those who did not. Previous study has shown that individuals who use smartphones with SMS and mobile internet features are highly susceptible to mobile addiction and severe symptoms as a result of this addiction compared to those who do not use these smartphones.^17^ In a 2013 study it was found that using cell phone communication methods such as messaging, social media, and e-mail lead to higher rates of problematic use.^18^ Although there may be studies among various populations, most students today use their cell phones to access the internet and social media platforms. This application-oriented use may increase the rate of problematic use.

In the study group, students who had spent most of their lifetime in a district or village demonstrated 2.09 times more problematic use of cell phones compared to students who spent it in a city. Students living in rural areas have limited access to recreational activities such as the cinema, theater and social facilities that they can attend in their leisure time; as a result, they may spend their free time on their cell phones and display higher rates of problematic use.

There is a mild, significant, positive correlation between the PU and LSAS scores of the students participating in the study. Similar to the results of our research, a study conducted in our country’s university showed a positive correlation between problematic internet use, loneliness and anxiety levels^16^. Moreover, there are studies showing that individuals who use their cell phones problematically frequently exhibit psychological problems such as depression, anxiety and decreased life satisfaction.^19^,^20^ Studies in the literature whose topics are similar to that of our research have reported similar results.

No significant relationship was found between the PU and EAT scores in the study group. Although no study in the literature has examined the relationship between the eating attitude test and cell phone addiction or problematic use of cell phones, there are studies analyzing its relationship with internet addiction. In our country, a study conducted in 2014 found no significant correlation between the EAT and internet addiction, similar to our study; however, the study reported a significant positive correlation between body mass index and internet addiction.^21^ A cohort study conducted in China with 1252 high school and university participants reported that females with internet addiction demonstrated significantly higher eating attitude test scores compared to females without addiction.^22^ Since an increase in body mass index is certainly an indication of obesity or high body weight, an increase in the problematic use of cell phone score may be associated with an increase in body mass index.

In addition to the detrimental effects of problematic use alone, mobile phones carry significance because they can contribute to the occurrence of social anxiety and eating disorders, as was also found in our study. Young university students should be encouraged to join social communities so that they may take a break from technology and spend their leisure time developing meaningful relationships. Thus, the young individual who begins contributing to the community can be more integrated with society and limit his/her mobile phone use, where he/she alleviates social loneliness.

### Authors’ Contribution:

**AG;** designed, did data collection, manuscript writing, final approval of manuscript.

**AO;** did statistical analysis, manuscript writing and editing manuscript.
